# Psychometric validation of the Chinese Version of the stimulant relapse risk scale (SRRS) in patients with methamphetamine use disorder

**DOI:** 10.1186/s13011-024-00616-8

**Published:** 2024-07-08

**Authors:** Jing-Shu Lin, Yasukazu Ogai, Chun Lin, Hu-Ming Chang, Yi-Chia Wu, Ming-Chyi Huang, Su-Chen Fang

**Affiliations:** 1 Department of Addiction Sciences, Taipei City Psychiatric Center, Taipei City Hospital, Taipei, Taiwan; 2 Center for Neuropsychiatric Research, National Health Research Institutes, Miaoli, Taiwan; 3 Social Psychiatry and Mental Health, Faculty of Medicine, University of Tsukuba, Tsukuba, Japan; 4 Addictive Substance Project, Tokyo Metropolitan Institute of Medical Science, Tokyo, Japan; 5 Department of Psychiatry, School of Medicine, College of Medicine, Taipei Medical University, Taipei, Taiwan; 6 Psychiatric Research Center, Taipei Medical University Hospital, Taipei, Taiwan; 7 Department of Nursing, Mackay Medical College, No.46, Section 3, Zhongzheng Road, Sanzhi District, New Taipei City, 252 Taiwan; 8 Department of Psychiatry, Taipei City Psychiatric Center, Taipei City Hospital, No. 309, Songde Road, Xinyi District, Taipei City, 110 Taiwan; 9 Kunming Prevention and Control Center, Taipei City Hospital, Taipei, Taiwan

**Keywords:** SRRS, Psychometric properties, Instrument, Validity, Methamphetamine

## Abstract

**Background:**

Evaluating the risk of relapse is a pivotal step in the treatment of patients with methamphetamine use disorder (MUD). The 30-item Stimulant Relapse Risk Scale (SRRS) was originally developed in Japan to meet the demand. This study examined the reliability, validity, and factor structure of the Chinese version of the SRRS for patients with MUD.

**Methods:**

247 patients with MUD self-rated the Chinese version of the SRRS. Cronbach’s alpha coefficients and inter-item correlation analysis were used to assess the internal consistency reliability. Construct validity was determined through confirmatory factor analysis (CFA), and concurrent validity was examined using the visual analogue scale (VAS) for drug craving and the severity of dependence scale (SDS). We followed the participants for 1 year and assessed the predictive validity based on the correlation of the scores of the Chinese version of the SRRS with the relapse rate within 3, 6, and 12 months of follow-up.

**Results:**

CFA revealed satisfactory model fit estimates for the 22-item Chinese version of the SRRS that consisted of four subscales. The four-factored 22-item Chinese version of the SRRS had adequate internal consistency with Cronbach’s alphas ranging from 0.76 to 0.92. The 22-item Chinese version of the SRRS scores were significantly correlated with the VAS and SDS scores as well as the relapse rate within 3, 6, and 12 months, indicating good concurrent and predictive validity of this scale. The receiver operating characteristic curve revealed a cutoff score of 40 could discriminate between participants with (SDS score ≥ 4) and without (SDS score < 4) methamphetamine dependence (area under the curve = 0.71, *p* < 0.01).

**Conclusions:**

The 22-item Chinese version of the SRRS that consists of four subscales is a valid and reliable instrument to assess the relapse risk in patients with MUD.

**Supplementary Information:**

The online version contains supplementary material available at 10.1186/s13011-024-00616-8.

## Introduction

Amphetamine-type stimulants are a group of psychostimulant drugs that are chemical derivatives of the parent compound alpha methylphenethylamine. Among these drugs, methamphetamine accounts for 95% of all manufactured amphetamine-type stimulants [1] and is the second most widely used illegal drug in the world [2, 3]. Methamphetamine use disorder (MUD) is a relapsing disorder that poses a significant threat to public health worldwide. According to the World Drug Report, In 2021, an estimated 36 million people aged 15–64 years old are estimated to have used amphetamines (methamphetamine and amphetamine) worldwide [4]. Long-term use of methamphetamine has been associated with adverse consequences including psychosis, depression, suicidality [5], cardiovascular and cerebrovascular diseases [6], sexually transmitted infection [7], and overdose [7, 8].

Because methamphetamine is highly addictive, MUD is associated with a high risk of relapse even after treatment. Studies involving patients with MUD who sought treatment have revealed post-treatment relapse rates ranged from 61% [9] to 69% ` [10]. Relapse is the major obstacle in substance use treatment and the most common outcome measure in substance-related research [11]. Evaluating the risk of relapse is a pivotal step in treating patients with MUD. This evaluation enables the design of a tailored intervention for patients with different risk levels, optimizing the efficient allocation of care services. Therefore, the development of a reliable tool to assess the relapse risk of those with MUD is indispensable in clinical service. Assessment of the relapse risk is complicated because various dimensions may jointly influence the risk of relapse, such as craving [12], stress [13], negative emotional states, and cognitive function [14, 15], and thus underscoring the necessity to incorporate multiple factors when developing tools to assess relapse risk for individuals who use substance. So far, only limited instruments have been developed to achieve this goal. The Advanced WArning of RElapse (AWARE) scale is a single-factored tool that has been validated to predict the relapse risk of substance use at 1-, 3-, and 6-months post-discharge [16]. Nevertheless, that study has theoretical limitation as the scale assesses reuse any substances, such as alcohol, marijuana, heroin, and cocaine, without accounting for the varying pharmacological effects, reasons for use, and user characteristics associated with each substance. Another recently published scale, the Risk of Relapse Assessment Scale (RRAS) was also demonstrated to be a good instrument measure the risk of relapse to methamphetamine use. The 16-item RRAS consisted of 3 dimensions, included ‘craving for methamphetamine’, ‘social recognition’, and ‘attitude towards methamphetamine’. Despite favorable construct validity and reliability of the RRAS, the predictive validity has not been assessed and validated [17]. With reference to the Marijuana Craving Questionnaire [18], Ogai et al. (2007) developed the Stimulant Relapse Risk Scale (SRRS) in Japan to measure the multiple facets of risk of stimulant reuse [19]. The SRRS is a multidimensional self-rated scale comprising 30 items in five dimensions: (1) anxiety and intention to use the drug (AI), (2) emotionality problems (EP), (3) compulsivity for the drug (CD), (4) positive expectancies and lack of control over the drug (PL), and (5) lack of negative expectancy for the drug (NE). The internal consistency, concurrent validity, and predictive validity of these subscales have been demonstrated to be adequate; thus, the SRRS is appropriate for predicting the relapse of stimulant use disorder, including MUD.

In Taiwan, methamphetamine surged to become the major illegal drug use since its epidemic in early 1990s. The recent two waves of National Survey of Substance Use respectively held in 2014 [20] and 2018 [21] reveal that methamphetamine remains the predominant illegal drug of use in Taiwan. Given the prevalence of methamphetamine use, it is imperative to have a reliable instrument to assess the relapse risks in people who use methamphetamine. Therefore, in the present study we aimed to examine the psychometric properties of the Chinese version of the SRRS by investigating its reliability, factor structure, and validity. In addition, we followed patients seeking MUD treatment for one year to explore the potential of the Chinese version of the SRRS to predict relapse during 3, 6, and 12 months.

## Materials and methods

### Participants and study design

This was a prospective study conducted in Taiwan. The study complied with the ethical standards described in the Declaration of Helsinki and received approval from the Institutional Review Board of the Taipei City Psychiatric Center (TCPC; ITCHIRB-10,810,018) before the study began. A thorough description of the study, encompassing its purpose, procedures, potential risks, benefits, the voluntary nature of their involvement, as well as the right to privacy protection and withdrawal from the study at any time, was provided prior to obtaining written informed consent for participation.

A total of 247 individuals who used methamphetamine and sought addiction treatment at the Department of Addiction Sciences, Taipei City Psychiatric Center of Taipei City Hospital, were recruited from January 1, 2016, to August 30, 2018. The treatment program was implemented in the outpatient setting for 12 months and was based on a standardized protocol that adopts a multicomponent approach involving a motivational interview, psychotherapy aimed at relapse prevention, and regular urine toxicology tests for methamphetamine and other illicit drugs. The inclusion criteria of our study were as follows: (1) age ≥ 18 years; (2) fulfillment of the *Diagnostic and Statistical Manual of Mental Disorders*,* 5th Edition* criteria for stimulant use disorder, amphetamine-type substance (methamphetamine), as verified by at least one board-certified psychiatrist; (3) with methamphetamine as the main drug of use in the past. The exclusion criteria were: (1) having a history of other substance use disorder, except tobacco; (2) inability to read Chinese and provide written informed consent. Research assistants were trained for collecting demographic data, administering the Chinese version of the SRRS, and conducting other psychological assessments.

### Instruments and measures

#### The Chinese version of the SRRS

Consistent with the standard forward–backward translation procedure (WHO, www.who.int/substance_abuse/research_tools/translation/en/), the Chinese version of the SRRS was first translated from its original version (i.e., the SRRS) in Japanese [19] into Chinese by two independent translators. Inconsistencies identified between the two translators were addressed through discussion involving another unbiased, bilingual translator (one of the researchers). Following this, back-translation was carried out by the two independent translators. To verify the translation accuracy, the back-translated version underwent discussion with the original developer [19]. The pre-final version of the translated scale was modified and reviewed by several mental health experts until a consensus was reached on all items, resulting in the final version of the scale.

The Chinese version of the SRRS comprises 30 items distributed among five subscales and features five additional items (Items 4, 11, 13, 15, and 26) as supplementary questions for the construct *insight into mental condition* (Supplementary Table 1). The five subscales and the respective items are as follows: (1) anxiety and intention to use the drug (AI) (Items 1, 2, 6, 12, 22, 27, 33, and 35; total = 8); (2) emotionality problems (EP) (Items 3, 5, 7, 10, 16, 19, 23, and 25; total = 8); (3) compulsivity for the drug (CD) (Items 8, 28, 31, and 3; total = 4); (4) positive expectancies and lack of control over the drug (PL) (Items 18, 20, 24, 29, 30, and 32; total = 6); and (5) negative expectancy for the drug (NE) (Items 9, 14, 17, and 21; total = 4). We examined the inner structure without the supplementary questions by using factor analysis. All items are listed in Supplementary Table 1. Item scores range from 1 (*strongly disagree*) to 5 (*strongly agree*), except for five items (Items 9, 12, 14, 17, and 21), which were reversely coded.

#### Measurements for concurrent validity

To evaluate the concurrent validity, we explored the correlation of the Chinese version of the SRRS scores with the severity of craving and methamphetamine dependence respectively. The severity of craving was measured by visual analogue scale (VAS) of the subjective intensity of craving for methamphetamine. Participants responded to the question “How strong is your craving for methamphetamine?” on a 0–100 scale, with “no craving” and “extreme craving” being the anchors on the extreme left and right of the scale, respectively [22, 23]. With regard to the severity of methamphetamine dependence, participants self-administered the 4-item Severity of Dependence Scale (SDS), the Chinese version of which was previously validated [24]. A score of ≥ 4 on the scale is indicative of the presence of severe methamphetamine dependence [25]. We also determined the potential of the Chinese version of the SRRS to distinguish the participants with or without a possible presence of methamphetamine dependence, analogous to the severe form of MUD.

#### Measurements for predictive validity

To assess predictive ability of the baseline scores on the Chinese version of the SRRS for relapse risk, the records of relapse within the subsequent 3, 6, and 12 months of follow-up were collected. Relapse was operationally defined as “any positive urine toxicology result for methamphetamine or verbal report of methamphetamine use during the follow-up period.”

### Statistical analysis

Calculated Cronbach’s alpha coefficients and inter-item correlation matrix analysis were used to assess the internal consistency reliability of the total Chinese version of the SRRS and its subscales. Cronbach’s alpha value of ≥ 0.7 [26] and item–total correlation of > 0.4 [27] were considered statistically acceptable.

Confirmatory factor analysis (CFA) was conducted to examine construct validity based on the original subconstructs suggested by [19]Ogai et al. (2007) using the CALIS procedure in SAS version 9.4 (SAS Institute, Cary, NC) CFA was performed using the robust maximum likelihood estimator method to determine the goodness of fit. Five indices were analyzed to evaluate the model fit: chi-square (χ2), normed chi-square (CMIN/DF ≈ 2) [28], adjusted goodness of fit index (AGFI) > 0.8 [29]; comparative fit index (CFI) > 0.9 [30]; standardized root mean square residual (SRMR) < 0.06 [23]; and root mean square error of approximation (RMSEA) < 0.08 [22]. Lagrange multiplier (LM, called a modification index in AMOS) estimates of item loadings of different factors were assessed to identify complex items and potential ways to improve the model [31]. The CFA model was modified until all the model fit indices met the established criteria. Notably, all deletions and modifications were incorporated one by one, and the CFA model was respecified following each modification.

The concurrent validity was determined by Pearson’s correlation analysis of the Chinese version of the SRRS with VAS and SDS scores.The receiver operating characteristic (ROC) curves with Youden’s index were used to determine the optimal cutoff points for the Chinese version of the SRRS scores in participants with high or low methamphetamine dependence severity based on SDS ≥ 4 and < 4, respectively. An area under the ROC curve (AUC) of 0.7 and 0.9 indicates moderate accuracy, and an AUC above 0.9 indicates high accuracy (Fischer, 2003). Finally, to estimate the predictive validity, we calculated the correlation of the Chinese version of the SRRS scores with the relapse rate with 3, 6, 12 months of follow-up. The data of participants who dropped out during the follow-up period were excluded from Pearson correlation model.

All statistical analyses were conducted using SAS (version 9.4). Descriptive statistics were calculated for all variables (i.e., medians and interquartile ranges [IQRs] for continuous variables and percentages for categorical variables).

## Results

### Participant characteristics

A total of 247 patients were recruited. The mean age of the participants was 34 (IQR = 30–41) years, with men being predominant (*n* = 227; 90.7%). Most of the participants were unmarried (73.6%) and employed (89.07%). The average duration of methamphetamine use was 3 years. During the follow-up period of 3, 6, and 12 months, 56 (22.67%), 75 (30.36%), and 94 (38.05%) participants experienced relapse, respectively. Table 1 summarizes the other demographic and methamphetamine use characteristics including detailed relapse rate and drop-out rate.

**Table 1:** Baseline demographic and clinical characteristics of participants (*N* = 247)

	Overall (*N* = 247)
Age in years, median (IQR)	37 (30–44)
Sex, n (%) Male Female	
	227 (91.9)
	20 (8.1)
Married, n (%) unmarried married other	
	179 (73.6)
	26 (10.61)
	40 (16.33)
Education, n (%)	
Less than or equal to high school education	112 (45.34)
High than high school senior	135 (54.66)
Employed, n (%)	
Yes	220 (89.07)
No	27 (10.93)
***Methamphetamine use-related variables***	
Years of using methamphetamine, median (IQR)	3 (1–8)
Age of first methamphetamine use, median (IQR)	30 (23–37)
SDS score^a^, median (IQR)	4 (2–6)
≥ 4, n (%)	116 (47.00)
< 4, n (%)	131 (53.00)
VAS for craving (score), median (IQR)	10 (0-26.5)
Relapse rate, n (%)	
Within 3 months	56 (22.67)
Within 6 months	75 (30.36)
Within 12 months	94 (38.05)
Drop-out rate, n (%)	
Within 3 months	5 (2.02)
Within 6 months	12 (4.86)
Within 12 months	21 (8.5)

^a^SDS ≥ 4 indicates the possibility of methamphetamine dependence.

Abbreviations: IQR: interquartile range; SDS: Severity of Dependence Scale; VAS: visual analogue scale

### Reliability analysis of internal consistency

Table 2 presents the item characteristics, descriptive statistics, and internal consistency reliability of the Chinese version of the SRRS. The Cronbach’s alpha coefficient for the total score of these items was 0.92, with item coefficient of “Cronbach’s alpha if item deleted” ranging from 0.91 to 0.92. The item–total correlations ranged from 0.02 to 0.73. Because of poor internal consistency, with item–-total correlations was less than 0.4 for each [27], four items (items 9, 14, 17, and 21) on the NE subscale were discarded. The Cronbach’s alpha coefficients for AI, EP, CD, and PL subscales ranged from 0.80 to 0.86, indicating satisfactory internal consistency across dimensions. A detailed overview of the aforementioned item characteristics and item–subscale total correlations are provided in Table 3. The remaining 26 items, representing a four-factor structure, were further analyzed in the CFA model.

**Table 2:** Item characteristics, item–total correlations, and Cronbach’s alpha values if an item is deleted in the Chinese version of the SRRS (*N* = 247)

Item	Mean (SD)	Cronbach’s Alpha	Item-total correlation	Cronbach’s Alpha if Item Deleted
Item 1	2.85 (1.11)		0.57	0.92
Item 2	2.57 (1.11)		0.73	0.91
Item 3	1.93 (0.96)		0.48	0.92
Item 5	2.39 (1.09)		0.52	0.92
Item 6	2.89 (1.29)		0.51	0.92
Item 7	2.49 (1.13)		0.57	0.92
Item 8	1.54 (0.83)		0.51	0.92
Item 9	2.53 (1.21)		0.25	0.92
Item 10	2.00 (1.03)		0.60	0.92
Item 12	1.94 (1.06)		0.54	0.92
Item 14	3.18 (1.46)		0.02	0.92
Item 16	2.49 (1.22)		0.58	0.92
Item 17	2.17 (1.17)		0.36	0.92
Item 18	2.08 (1.08)		0.70	0.91
Item 19	2.93 (1.32)		0.42	0.92
Item 20	2.06 (1.08)		0.70	0.91
Item 21	2.48 (1.31)		0.17	0.92
Item 22	1.43 (0.67)		0.63	0.92
Item 23	1.68 (0.82)		0.67	0.91
Item 24	1.84 (0.94)		0.71	0.91
Item 25	2.60 (1.22)		0.50	0.92
Item 27	1.53 (0.74)		0.71	0.91
Item 28	1.39 (0.67)		0.58	0.92
Item 29	1.76 (0.94)		0.72	0.91
Item 30	1.66 (0.88)		0.70	0.91
Item 31	1.29 (0.63)		0.52	0.92
Item 32	2.21 (1.23)		0.62	0.92
Item 33	1.55 (0.73)		0.64	0.92
Item 34	1.25 (0.58)		0.53	0.92
Item 35	1.38 (0.72)		0.59	0.92
Total	72.30 (18.81)	0.92		

Items 4, Item 11, Item13, Item 15, and Item 26 are supplementary questions that were not included in the analysis

**Table 3:** Item characteristics, item–total correlations, and Cronbach’s alpha values if an item is deleted in the Chinese version of the SRRS (*N* = 247)

Subscale	No. of items (score)	Mean (SD)	Cronbach’s Alpha of factor	Item- subscale total correlation	Cronbach’s Alpha if Item Deleted
Al	8 (8–40)	16.14 (5.00)	0.83		
Item 1		2.85 (1.11)		0.70	0.82
Item 2		2.57 (1.11)		0.83	0.79
Item 6		2.89 (1.29)		0.63	0.83
Item 12		1.94 (1.06)		0.60	0.82
Item 22		1.43 (0.67)		0.61	0.81
Item 27		1.53 (0.74)		0.74	0.79
Item 33		1.55 (0.73)		0.69	0.80
Item 35		1.38 (0.72)		0.58	0.82
EP	8 (8–40)	18.5 (5.67)	0.80		
Item 3		1.93 (0.96)		0.49	0.80
Item 5		2.39 (1.09)		0.68	0.77
Item 7		2.49 (1.13)		0.74	0.76
Item 10		2.00 (1.03)		0.66	0.77
Item 16		2.49 (1.22)		0.71	0.76
Item 19		2.93 (1.32)		0.61	0.79
Item 23		1.68 (0.82)		0.59	0.78
Item 25		2.60 (1.22)		0.65	0.78
CD	4 (4–20)	5.47 (2.14)	0.80		
Item 8		1.54 (0.83)		0.72	0.84
Item 28		1.39 (0.67)		0.78	0.76
Item 31		1.29 (0.63)		0.83	0.70
Item 34		1.25 (0.58)		0.83	0.69
PL	6 (6–30)	11.61 (4.76)	0.86		
Item 18		2.08 (1.08)		0.77	0.85
Item 20		2.06 (1.08)		0.74	0.86
Item 24		1.84 (0.94)		0.81	0.84
Item 29		1.76 (0.94)		0.82	0.83
Item 30		1.66 (0.88)		0.79	0.84
Item 32		2.21 (1.23)		0.73	0.86
Item 21		2.48 (1.31)		0.62	-0.39

Abbreviations: AI: anxiety and intention to use the drug; EP: emotionality problems; CD: compulsivity for the drug; PE: positive expectancies and lack of control over the drug

### Construct validity

**Fig. 1:**
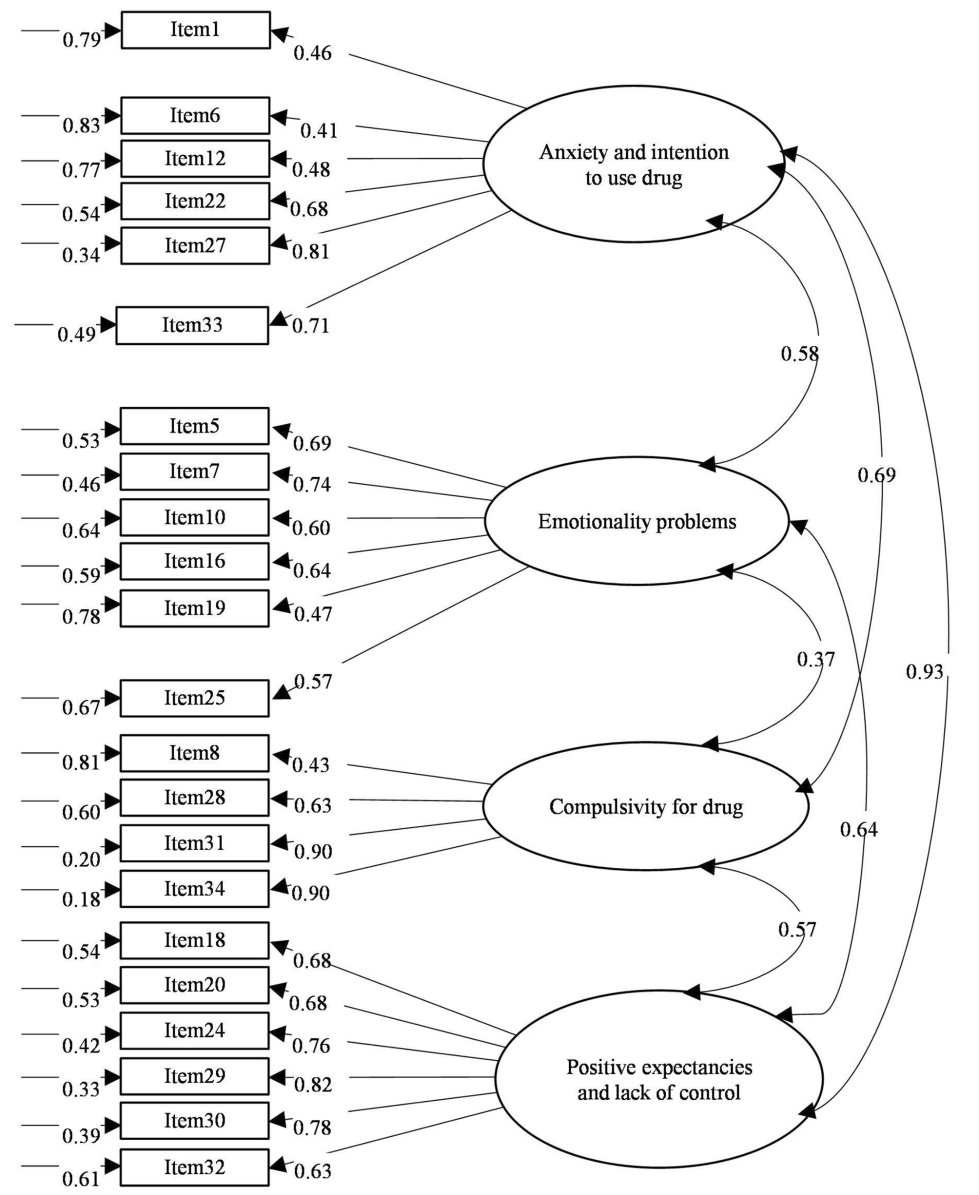
Structural model with factor loading, error variance, and correlations Single headed arrow reflects factor loading and error variance. Double headed arrows display correlation. Factor loading and correlation are presented as standardized estimates

CFA was used to examine the goodness of fit of the 26-item Chinese version of the SRRS (Supplementary Fig. 1). The goodness-of-fit indices revealed that the Chinese version of the SRRS did not fit the data well (normed χ2 = 2.18, AGFI = 0.80, CFI = 0.90, SRMR = 0.07, and RMSEA = 0.07). Four items (Items 2, 3, 23, and 35) had high LM scores, indicating cross-loadings on two or more factors. Hence, these four items were removed to maximize the item–remainder Cronbach’s alpha coefficients and factor loadings, yielding the final Chinese version of the SRRS composed of 22 items (Fig. 1). The modified 22-item Chinese version of the SRRS model fit the data well (normed χ2 = 1.93, AGFI = 0.84, CFI = 0.93, SRMR = 0.06, and RMSEA = 0.06). The CFA fit indices are presented in Table 4. Figure 1 illustrates the correlation matrices among the latent variables and factor loadings; all standardized factor loadings exceeded the threshold of 0.4, indicating that the item–total correlations of the Chinese version of the SRRS items were within acceptable ranges [32]. Regarding internal consistency, the subscales and total 22-item version had favorable Cronbach’s alpha coefficients, ranging from 0.76 to 0.92, respectively (Table 5)

**Table 4:** Confirmatory factor analysis fit indices (*N* = 247)

Model	χ 2	df	Normed chi-square	AGFI	CFI	SRMR	RMSEA (95% CI)
26-item							
Initial model	984.35	293	3.36	0.68	0.79	0.09	0.10 (0.09–0.10)
Modification model	623	285	2.18	0.80	0.90	0.07	0.07 (0.06–0.08)
22-item							
Initial model	541.39	203	2.66	0.77	0.86	0.08	0.08 (0.07–0.09)
Modification model	381.01	197	1.93	0.84	0.93	0.06	0.06 (0.05–0.07)

Abbreviations: χ2 = chi-square; df = degrees of freedom; AGFI = adjusted goodness of fit index; CFI = comparative fit index; SRMR = standardized root mean square residual; RMSEA = root mean square error of approximation

**Table 5:** Cronbach’s alpha of each subscale of the Chinese version of the SRRS and correlation with VAS, SDS, and relapse (*N* = 247)

	Cronbach’s Alpha		Correlation					
			VAS	SDS	3-month relapse rate	6-month relapse rate		12-month relapse rate
22-item Chinese version of the SRRS		0.92	0.59**	0.39**	0.21**	0.20**		0.15*
Subscale								
AI		0.76	0.63*	0.42**	0.22**	0.21**		0.15*
EP		0.78	0.36**	0.33**	0.14*	0.14*		0.10
CD		0.80	0.31**	0.15*	0.12	0.13*		0.08
PE		0.86	0.59**	0.32**	0.20**	0.18**		0.14*

Reliability was calculated according to Cronbach’s alpha

Concurrent validity was calculated according to the correlation of the 22-item Chinese version of the SRRS total score with 3-, 6-, and 12-month relapse, VAS scores, and SDS scores

Abbreviations: SDS: Severity of Dependence Scale; VAS: visual analogue scale; AI: anxiety and intention to use the drug; EP: emotionality problems; CD: compulsivity for the drug; PE: positive expectancies and lack of control over the drug

*P < 0.05

**P < 0.01

### Concurrent validity

**Fig. 2:**
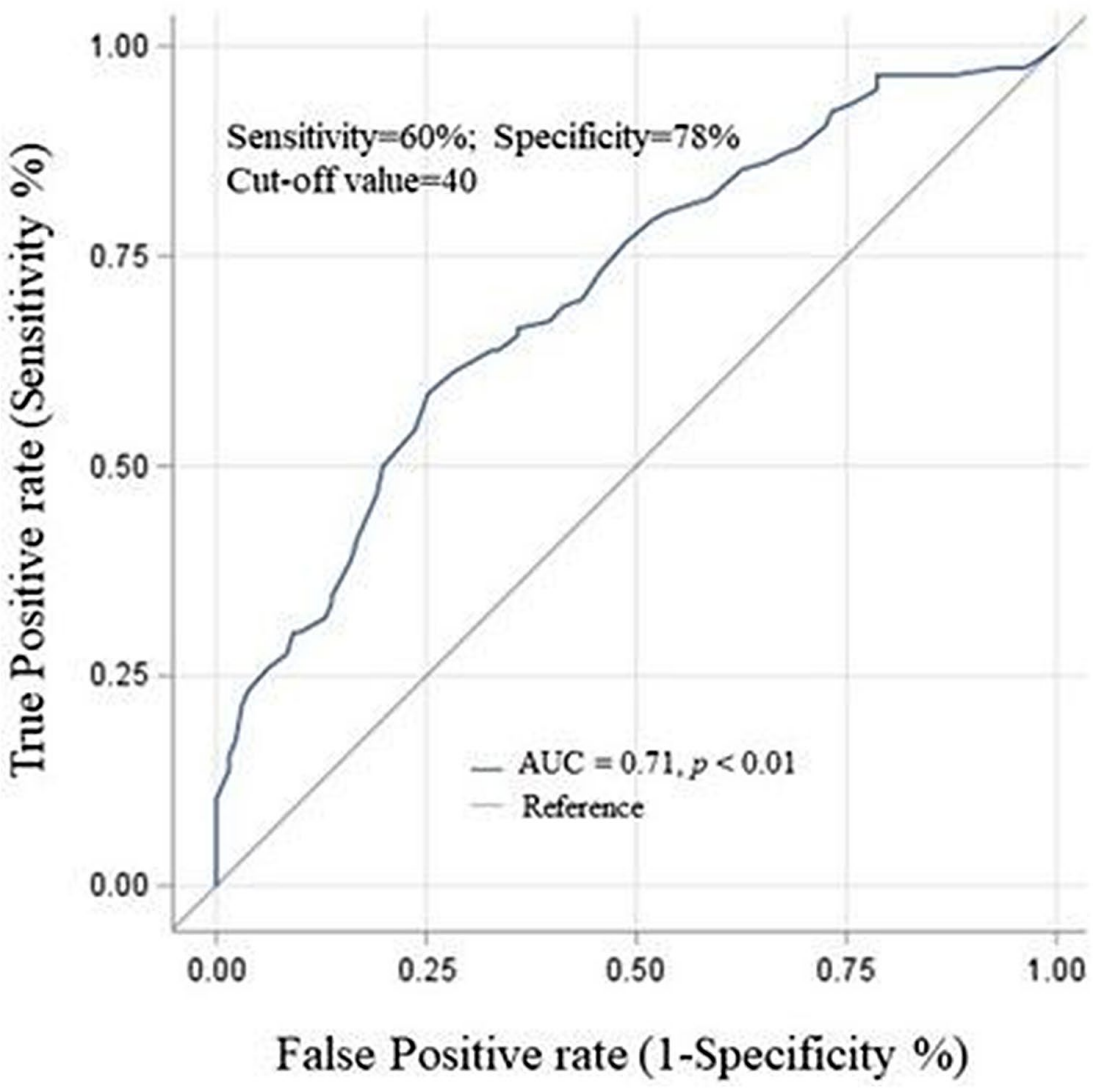
The ROC curve of the 22-item Chinese version of the SRRS to discriminate between participants with or without methamphetamine dependence Note: A cutoff score of 40 of the Chinese version of the SRRS yielded an AUC of 0.71, reaching 60% sensitivity and 78% specificity (*p* < 0.01)

The 22-item Chinese version of the SRRS and its subscales scores significantly correlated with the VAS (*r* = 0.59, *p* < 0.01 for the Chinese version of the SRRS total scores; *r* = 0.63 − 0.31, *p* < 0.01 for the subscales scores) and the SDS scores (*r* = 0.39, *p* < 0.01 for the Chinese version of the SRRS total scores; *r* = 0.42 − 0.15, *p* < 0.01–0.05 for the subscales scores), suggesting the Chinese version of the SRRS had favorable concurrent criterion validity (Table 5). ROC curve analysis revealed that the 22-item Chinese version of the SRRS had moderate accuracy to discriminate individuals with (SDS score of ≥ 4) and without (SDS score of < 4) methamphetamine dependence (estimate: 1.07; 95% confidence interval: 1.04**–**1.09), with an AUC of 0.71 (Fig. 2). Considering the maximum Youden’s *J* value, an optimal cutoff score of 40 could distinguish between patients with low and high methamphetamine dependence in the current analysis (Youden’s *J* = 1.38; sensitivity = 60%, specificity = 78%) (Supplementary Table 2).

### Predictive validity

Table 5 presents the relationships of the 22-item Chinese version of the SRRS scores with relapse rates during the 3-, 6-, and 12-month follow-up periods. Except CD subscale, the total and AI/PE subscale scores were significantly correlated with the relapse rate at all time points, with the strength of the correlation decreasing over time (*r* = 0.22 − 0.18, *p* < 0.01 at 3- and 6- month, but *r* = 0.14, *p* < 0.05 at 12-month). EP also exhibited a similar trend—the correlation with the relapse rate was weak positive significant initially (at 3 and 6 months) but diminished at longer follow-ups (at 12 months).

## Discussion

To the best of our knowledge, this is the first study to examine the factor structure, reliability, and validity of the Chinese version of the SRRS in patients with MUD. Our results indicated that the 22-item Chinese version of the SRRS with four subscales—AI, EP, CD, and PL— generally had satisfactory reliability and validity. The concurrent validity of the 22-item Chinese version of the SRRS and its four subscales was confirmed through its significant correlation with the VAS and SDS scores. The ROC curve results also revealed that with a cutoff score of 40, the 22-item Chinese version of the SRRS had a moderate accuracy to differentiate between individuals with high and low severity of methamphetamine dependence. Moreover, the significant correlation of the 22-item Chinese version of the SRRS scores with 3-, 6-, and 12-month methamphetamine relapse rates suggests the 22-item Chinese version of the SRRS has strong predictive validity. These observations collectively highlight that the present 22-item Chinese version of the SRRS may serve as a useful instrument for assessing the various aspects of relapse in patients with MUD.

The multidimensional structures of the Chinese version of the SRRS and the original SRRS have similarities and differences. In the initial reliability analysis of the Chinese version of the SRRS, the four items of the NE subscale (Items 9, 14, 17, and 21) demonstrated poor item–total correlations and were discarded, yielding a 26-item scale with a four-factor structure. This structure differs from that of the original Japanese version of SRRS, which contains five factors. The poor Cronbach’s alpha value for this subscale in our study is consistent with that obtained for the original version (0.545) [19]; that is, the internal consistency of this subscale was insufficient in both studies. The four items that constitute the NE subscale measure the lack of negative expectancy, referring to the influence of an individual’s expectations of substance-related outcomes on the initiation and maintenance of substance use [19, 33, 34]. Positive expectancies about substance use may increase the risk of relapse, whereas negative expectancies decrease the risk [33, 34]. However, evidence regarding the impact of negative expectancies on substance-taking behavior is limited [33, 34]. In addition, Item 14 (“I am afraid of hallucinations due to drug use”) and Item 17 (“I would not be able to control myself if I use the drug”), which previously formed a part of the NE subscale, were reverse-scored items. Reverse-scored items, designed to prevent a response bias, may lead to respondent confusion. In Asian culture, “not be able to control” may imply either a disinhibition effect secondary to drug use or poor control of personal impulsivity, and an experience of hallucination may imply “going crazy” or having an existing mental disorder. The potential for item misinterpretation and effect of societal stigma may result in unreliable answers to these two items.

Based on CFA results, four items (Items 2, 3, 23, and 35) were further discarded, yielding the final 22-item Chinese version of the SRRS, which is different from the original 30-item SRRS. The aforementioned four items exhibited high LM scores, indicating the items carried cross-loadings on two or more factors. For example, Items 2 and 35, which were derived from the AI subscale, and Item3 from the EP subscale exhibited substantial cross-loadings on non-targeted CD factor. The cross-loadings were also observed for Item 23, which was derived from the EP subscale but exhibited cross-loadings on non-targeted AI factors. The results for Item 2 (“There are times I want to use the drug “) and Item 35 (“Even though I know I will be arrested, I would use the drug”) could be explained based on the multidimensional manifestations of drug craving, which might result in divergent interpretation among individuals. The development process of craving has been conceptualized from distinct theoretical constructs; no conceptual construct can fully depict the complex phenomenon of craving. For instance, compulsivity, expectancy, anxiety about relapse, and intention to use drugs have all been proposed as key components of craving. In classical learning models and the obsessive-compulsive theory of substance dependence, which are characterized by conditioned reinforcement and compulsive drug use [12, 18, 35], craving is regarded as a conditioned and anticipatory response to drug-related stimuli such as drug-like or withdrawal-like effects [12, 36]. However, from the cognitive-behavioral perspective, craving is mediated by the anticipated effect of taking the drug [12, 36]. As a result, craving can take various forms, including liking, wanting, urges, desires, need, intention, expectancy, anticipation, or compulsion to use [19, 36]. According to obsessive–compulsive theory, the items on the CD subscale may reflect the craving phenomenon whereas the items on the AI subscale may reflect anxiety about relapse, anticipation of relapse, retrial of memory about using drugs, and intention to use drugs. Item 2 (“There are times I want to use the drug”) and Item 35 (“Even though I know I will be arrested, I would use the drug’) refer to the intention to use the drug in response to cravings and even further compulsion to use drug, despite harmful consequences. On the other hand, Item 3 and Item 23 were originally categorized into EP subscale, which reflects “common feelings and moods, especially negative emotional states observed before relapse of drug use”. However, for Item 3 (“I feel a constant need to put something in my mouth”), respondents might misinterpret “need to put something in my mouth” as the need or craving for the drug by ingestion or inhalation, rather than emotionality problems. “Regarding Item 23 (“I cannot control my feelings”), a notable cross-loading was observed, indicating a high correlation with AI, EP, and PE factors. In the Chinese context, the word “feeling” is vague and struggles to convey specific feelings or emotions. Moreover, many Asian cultures value conformity to norms, emotional self-control, and collectivism [37]. The display of emotions, particularly psychological distress or aggression may be perceived as a sign of mental illness and/or personal weakness, leading to stigmatization [37]. Asian culture groups tend to have more difficulty than Western culture groups in identifying and communicating emotions [38]. Item 23 in the Chinese version of the SRRS appears to be challenging to define accurately, leading to ambiguity and overlap among patients with MUD in Tiawan.

We used the SDS and VAS to examine the concurrent validity of the 22-item Chinese version of the SRRS. The Chinese version of the SRRS and its four subscales exhibited significantly positive correlation with the SDS and weak positive correlation with VAS scores. Furthermore, a cutoff score of 40 of the Chinese versions of the SRRS displayed the most favorable sensitivity and specificity in distinguishing individuals with and without methamphetamine dependence (i.e. severe MUD). The highest value for the Youden’s *J* statistic was 41 for the Chinese version of the SRRS; however, the difference between sensitivity and specificity was greater than that for the cutoff score of 40. Therefore, we suggest 40 as the optimal cutoff score for 22-item Chinese version of the SRRS for identifying the presence of severe MUD.

Regarding predictive validity, the total 22-item Chinese version of the SRRS and AI and PE subscale scores were significantly and positively correlated with the relapse rate within 3 and 6 months, but the correlation decreased at 12 month; this time-dependent reduction in the correlation over time was also observed in the EP and CD subscales. These results are similar to those obtained for the original version of the SRRS [19]. Research has revealed that the highest relapse rate for MUD is observed early in treatment, in particular within the first 6 months [9], suggesting that the risk of relapse might decline with time. In our study, we also found that the relapse rate was the highest in the first 3 months and decreased gradually during the 6- and 12-month follow-up periods. The Chinese version of the SRRS, therefore, may have the most favorable predictive value in the early stage of the treatment when the relapse risk is higher. The 22-item Chinese version of the SRRS also share certain similarities with previously validated measures of relapse risk such as the AWARE scale and the RRAS. The 22-item Chinese version of the SRRS and the AWARE scale both encompass some symptoms of negative affection and passivity. On the other hand, the nature of RRAS subscale ‘craving for methamphetamine’ is akin to the CD and AI subscales of the Chinese version of the SRRS. Additionally, “attitude towards methamphetamine” subscale of RRAS also implies expectations related to substance use, mirroring the PL subscale. On the contrary, the Chinese version of the SRRS did not assess social context like the ‘social recognition’ subscale in the RRAS.

This study has some limitations. First, the sample is not representative in terms of sex, with 90% of the respondents being men. Nevertheless, the predominance of men is consistent with a previous national survey in our country, which also reported that males using illegal drugs such as methamphetamine exceed 90% [20]. Second, we focused only on methamphetamine as the primary abused substance in this study. Although the total and subscale scores of the original SRRS did not differ significantly across stimulants such as methamphetamine, methylphenidate, and 3,4-methylenedioxymethamphetamine [19], the reliability and validity of the Chinese version of the SRRS in individuals who use other stimulants should be tested in the future. Third, we did not evaluate the psychiatric comorbidities, which are common in individuals with MUD [39]. Therefore, we are not able to understand the potential impact of these comorbidities on the psychometric properties of C-SRRS. Fourth, the Chinese version of the SRRS has a weak positive correlation with the relapse rates. A further study was needed to validate this result.

In conclusion, we demonstrated that the 22-item Chinese version of the SRRS is a reliable and valid instrument with favorable multidimensional psychometric properties for assessing the risk of relapse in patients with MUD.

### Electronic supplementary material

Below is the link to the electronic supplementary material.

Supplementary Fig. 1. Structural model with factor loading, error variance, and correlations.
Single-headed arrows indicate factor loading and error variance. Double-headed arrows display correlation. Factor loading and correlation are presented as standardized estimates.

Supplementary Material 2

## Data Availability

No datasets were generated or analysed during the current study.

## Abbreviations

SRRS: Stimulant Relapse Risk Scale
MUD: Methamphetamine use disorder
CFA: Confirmatory factor analysis
VAS: Visual analogue scale
SDS: The severity of dependence scale
RRAS: The Risk of Relapse Assessment Scale
AI: Anxiety and intention to use the drug
EP: Emotionality problems
CD: Compulsivity for the drug
PL: Positive expectancies and lack of control over the drug
NE: Lack of negative expectancy for the drug
AGFI: adjusted goodness of fit index
CFI: Comparative fit index
SRMR: Standardized root mean square residual
RMSEA: Root mean square error of approximation
LM: Lagrange multiplier
ROC: The receiver operating characteristic curves
AUC: Area under the ROC curve
