# Supplementary material for: Psychometric validation of the Chinese Version of the stimulant relapse risk scale (SRRS) in patients with methamphetamine use disorder
Source: Subst Abuse Treat Prev Policy. 2024 Jul 8;19:34. doi: 10.1186/s13011-024-00616-8 (PMC11232344; doi:10.1186/s13011-024-00616-8)
Supplement: Supplementary file 1 — Supplementary Fig. 1. Structural model with factor loading, error variance, and correlations. Single-headed arrows indicate factor loading and error variance. Double-headed arrows display correlation. Factor loading and correlation are presented as standardized estimates. [file 13011_2024_616_MOESM1_ESM.docx]

Supplementary Table 1. The wording of items on the SRRS.

| Item | Item description |
| --- | --- |
| Item 1 | The feeling I used to have while using the drug sometimes comes back |
| Item 2 | There are times I want to use the drug. |
| Item 3 | I feel a constant need to put something in my mouth |
| Item 4 | I can stop using the drug by myself. (R) |
| Item 5 | I am annoyed by words from others. |
| Item 6 | I am anxious about reusing the drug. |
| Item 7 | I am irritated. |
| Item 8 | I would do almost anything in order to use the drug |
| Item 9 | I feel easier than before. (R) |
| Item 10 | I am not motivated to do anything. |
| Item 11 | I would be fine without the drug. (R) |
| Item 12 | Thinking about my family, I can no longer use the drug. (R) |
| Item 13 | I have already recovered from drug abuse. (R) |
| Item 14 | I am afraid of hallucinations due to drug use. (R) |
| Item 15 | I am confident that I would not use the drug again. (R) |
| Item 16 | I feel lonely. |
| Item 17 | I would not be able to control myself if I use the drug. (R) |
| Item 18 | If someone holds the drug under my nose, I would not be able to refuse it. |
| Item 19 | I am anxious about my future. |
| Item 20 | I would use the drug if I am alone. |
| Item 21 | If I use the drug, it would badly influence my job. (R) |
| Item 22 | If my friend gives me the drug, I would use it even in the hospital. |
| Item 23 | I cannot control my feeling. |
| Item 24 | If the drug is placed in front of me, I would use it. |
| Item 25 | I feel tired due to impatience. |
| Item 26 | I think I am an addict. |
| Item 27 | If I have a large sum of money, I want to buy the drug. |
| Item 28 | I would do anything to get money for the drug. |
| Item 29 | If I use the drug, I would be less nervous. |
| Item 30 | If I use the drug, I would feel everything is going well. |
| Item 31 | I want the drug even if I have to steal. |
| Item 32 | If I use the drug, I would feel invigorated. |
| Item 33 | I will use the drug in near future. |
| Item 34 | I want to obtain the drug even by working illegally |
| Item 35 | Even though I know I will be arrested, I would use the drug. |

(R) = reverse-coded item

Supplementary Table 2. Sensitivity, specificity, and Youden’s index for the selected cutoff scores of the 22-item Chinese version of the SRRS.

| Cutoff score | Sensitivity | Specificity | Youden' J |
| --- | --- | --- | --- |
| 40 | 0.60 | 0.78 | 1.38 |
| 41 | 0.58 | 0.80 | 1.38 |
| 38 | 0.63 | 0.74 | 1.36 |
| 42 | 0.53 | 0.83 | 1.36 |
| 37 | 0.65 | 0.71 | 1.36 |
| 36 | 0.66 | 0.67 | 1.33 |
| 33 | 0.73 | 0.60 | 1.33 |
