# Supplementary figures and images for: Psychometric validation of the Chinese Version of the stimulant relapse risk scale (SRRS) in patients with methamphetamine use disorder
Source: Subst Abuse Treat Prev Policy. 2024 Jul 8;19:34. doi: 10.1186/s13011-024-00616-8 (PMC11232344; doi:10.1186/s13011-024-00616-8)

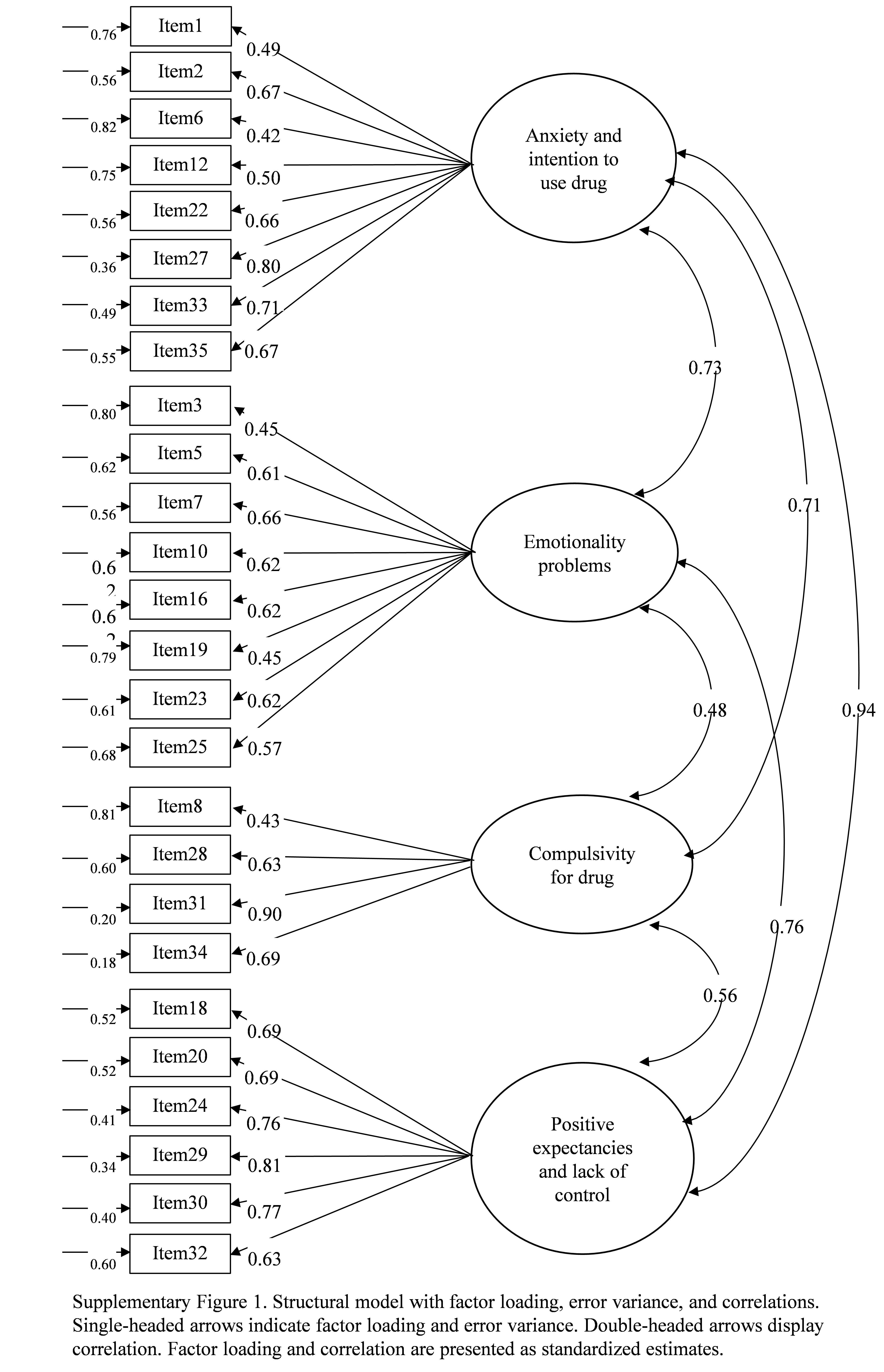

Supplement: Supplementary file 2 — Supplementary Material 2 [file 13011_2024_616_MOESM2_ESM.jpg]
